# Differences in intervention for patients with acute stroke according to the manpower of neurosurgeons

**DOI:** 10.1371/journal.pone.0319740

**Published:** 2025-03-10

**Authors:** Hyeong Sook Kim, Yun Seo Jang, Suk-Yong Jang, Chung Mo Nam, Eun-Cheol Park

**Affiliations:** 1 Department of Medical Fee Schedule, Health Insurance Review and Assessment Service, Wonju, Republic of Korea; 2 Department of Public Health, Graduate School, Yonsei University, Seoul, Republic of Korea; 3 Institute of Health Services Research, Yonsei University, Seoul, Republic of Korea; 4 Department of Preventive Medicine, Yonsei University College of Medicine, Seoul, Republic of Korea; Weill Cornell Medicine, UNITED STATES OF AMERICA

## Abstract

**Background and objectives:**

Stroke, a leading global cause of death, poses a substantial health burden. The incidence of stroke is high in an aging society. Appropriate healthcare resources are crucial for providing prompt interventions to patients with stroke. We investigated the factors associated with the choice between conservative and interventional treatments, including an analysis of the number of neurosurgeons required for interventional care, for patients with acute stroke.

**Methods:**

We utilized health insurance claims data from hospitals submitted to the Health Insurance Review and Assessment Service in 2018 and 2021. The data covered 60,661 patients with acute stroke admitted to the emergency room in tertiary or general hospitals. The number of hospital neurosurgeons was the key variable of interest; conservative and interventional treatments were the independent variables. Using a multi-level analysis, we identified the individual- and hospital-level factors associated with interventional treatment by constructing four models.

**Results:**

The odds of patients with hemorrhage and ischemic stroke receiving intervention were 0.60 [95% confidence interval (CI), 0.31–0.52] and 0.51 [95% CI, 0.39–0.65] times lower, respectively, in the group with fewer neurosurgeons. We categorized the number of neurosurgeons and indicated an association between a minimum of three neurosurgeons and stroke treatment.

**Conclusion:**

We demonstrated an association between individual- and hospital-level factors and the intervention for patients with different types of stroke. We predicted the number of neurosurgeons needed for intervention. These findings can be used for the efficient distribution and utilization of healthcare resources to improve public health.

## Introduction

Stroke, which encompasses all disorders and injuries in the brain, is classified as a cerebrovascular disease [1]. It includes ischemic stroke (blood clot or thrombus formation in a damaged blood vessel, blocking nutrient and oxygen supply to the brain), hemorrhagic stroke (resulting from blood vessel rupture in the brain, causing bleeding), and transient ischemic attacks (temporary disruption of the blood supply to the brain, resolving within 24 h without permanent damage) [1,2]. Acute stroke can lead to unpredictable or severe disabilities [3], thus necessitating proactive primary prevention. In case of stroke, appropriate treatment is necessary to minimize disability [4–6]. Particularly for the acute phase of stroke, timely and appropriate treatment is crucial within a certain time frame, termed the “golden time,” to maximize the chances of recovery [7,8]. The “golden time” refers to the critical period—up to 60 minutes for ischemic stroke to administer IV-tPA, and the urgent window for hemorrhagic stroke to control bleeding and intracranial pressure through surgical or endovascular interventions [8]. All necessary tests and treatments are administered within a few hours of patient arrival in the emergency room [9]. Therefore, the prompt judgment of a neurosurgeon who can determine the direction of diagnosis and treatment within this critical period, is essential [10].

Treatment for acute stroke includes general supportive care, prevention and treatment of internal complications, and thrombolysis [11,12]. The Third European Cooperative Acute Stroke study demonstrated the effectiveness of intravenous (IV) thrombolysis with tissue plasminogen activator (tPA) in ischemic stroke treatment; the earlier the administration, the better the effect. The efficacy decreases gradually and it is effective only up to 4.5 h of administration [13,14]. To shorten the time-to-treatment, integrated management involving medication, surgery, intervention, and rehabilitation is required [15,16]. Acute stroke symptoms can deteriorate or change rapidly, thus warranting close observation. These demands often necessitate the introduction of government stroke centers and allocation of medical and nursing staff [17–20].

However, in Korea, patients requiring urgent surgery because of conditions such as intracranial hemorrhage have died in the emergency room while waiting for a surgeon [21,22]. Thus, the demand for neurosurgeons will supposedly increase in Korea. However, the supply and deployment of health care professionals may not keep pace with this demand, particularly considering the domestic shortage of neurosurgeons. This drawback underscores the need for improvements in the domestic neurosurgical healthcare system and formulation of strategies to address medical professional shortage, emphasizing the importance of timely and adequate neurosurgical expertise.

Therefore, we aimed to investigate the association between the number of neurosurgeons and intervention methods in patients with acute stroke, which is a critical medical field directly related to national health and life.

## Methods

### Data

Data from the Health Insurance Review and Assessment Service (HIRA) claims records for medical expenses were used in this study. Initially, the government defined the evaluation period for assessing the appropriateness of acute stroke as a reference for the preceding 6 months. Based on this criterion, data from July 1, 2018 to December 31, 2018 and from July 1, 2021 to December 31, 2021 cover 6 months. The data used in this study consisted of encrypted personal identification numbers to ensure anonymity. The research procedures and content were approved for exemption from review by the Institutional Review Board of Health Insurance Review & Assessment Service (Approval Number: HIRAIRB-2023-085-001). Data were assessed from October 2023 to February 2024 for research purposes.

### Study population

We focused on patients aged ≥ 18 years admitted through the emergency room with a primary diagnosis of stroke (Korean Standard Classification of Diseases codes I60–I63) [23,24]. Medical specialties included neurology and neurosurgery, primarily involved in the treatment of patients who have had stroke (including brain specialties). The healthcare institutions were tertiary general hospitals and those categorized under the Appropriateness Evaluation for Acute Stroke Medical Institutions, which are typically hospitals where the patients with stroke seek treatment upon onset initially. The study population consisted of 107,138 individuals admitted between July 1, 2018 and December 31, 2021 and discharged by the end of the year. We excluded patients aged < 18 years and cases with unclaimed emergency care management fees (non-emergency cases).

We excluded (i) cases where admission and discharge occurred on the same day, (ii) cases with previous admissions in the first half of the year, (iii) instances of multiple admissions during the analysis (excluding the first admission in case of two or more admissions), and (iv) cases related to traumatic injuries and specialties, such as internal medicine, rehabilitation medicine, and emergency medicine. The final analysis focused on patients admitted only once without duplicate admissions in neurology and neurosurgery. This study included 60,661 participants from 345 institutions (S1 Fig).

### Variables

The dependent variable was categorized into conservative and interventional treatments, considering resource consumption and differences in patient conditions. Conservative treatment was defined as treatment involving antithrombotic therapy, neuro-protective drugs, or similar interventions. Interventional treatment was defined as treatment within the golden period of acute stroke, including surgery, interventional radiological procedures, and IV-tPA administration, and a combination of interventional radiological procedures and IV-tPA administration. An additional analysis was conducted to examine the impact of the number of neurosurgeons on the interventional treatment for acute stroke across four treatments: (1) surgery, such as decompressive craniectomy, cerebrospinal fluid drainage, and hematoma removal; (2) interventional radiological procedures, including endovascular thrombolysis; (3) IV-tPA administration after ischemic stroke; and (4) IV-tPA administration before endovascular thrombolysis and the concurrent administration of interventional radiological procedures.

It was categorized into terciles based on the neurosurgeon count in hospitals, as follows: Q1 (0–2), Q2 (3–4), and Q3 (≥5). Q1 and Q3 represent groups with a low and high number of neurosurgeons, respectively. The independent variables included individual-level factors, such as the sex, age, insurance eligibility, medical specialty, length of hospitalization, whether computed tomography or magnetic resonance imaging (MRI) was performed, treatment outcomes, Charlson Comorbidity Index, and year. Hospital-level factors were adjusted for the healthcare institution, establishment, and emergency medical institution types, medical institution location (metropolitan: Seoul and Gyeonggi, urban: Busan, Daegu, Gwangju, Incheon, Daejeon, Ulsan, and Sejong, rural: others), acute stroke evaluation grade, number of beds, annual stroke patient count, and number of neurologists.

### Statistical analysis

Descriptive statistics and frequency analyses were performed to determine the general characteristics of the patients. We compared the relevance of individual- and hospital-level variables based on the treatment for acute stroke. Moreover, we examined the frequencies, percentages, and P-values for each variable.

Furthermore, the differences in treatment based on the healthcare institution characteristics were analyzed using a multilevel analysis model that considered both individual- and hospital-level variables. The analysis was conducted using Generalized Linear Mixed Models in SAS through the Glimmix Procedure. A two-level multilevel logistic regression model was employed at the individual and hospital levels. Initially, a null model comprising only the constant term, a random-effects model comprising individual-level variables, and a mixed-effects model comprising both individual- and hospital-level variables were constructed. Model fit tests were performed; fixed and random effects at the individual and hospital levels, respectively, were analyzed.

Statistical analyses were performed using SAS 9.4 (SAS Institute Inc., Cary, NC, USA). NC. USA). The significance level for all analyses was analyzed using a two-tailed test. P-values < 0.05 were considered statistically significant.

## Results

Table 1 presents the analysis results of the individual-level characteristics of the patients. Of the 60,661 patients, 14,254 and 46,407 had hemorrhage and ischemic stroke, respectively. Approximately 46.2% and 15.8% of the patients with hemorrhage and ischemic stroke, respectively, underwent interventional treatment. The distribution of intracerebral hemorrhage and subarachnoid hemorrhage within hemorrhagic stroke and the differences in intervention methods for each are reported in S2 Table. Differences in the intervention methods demonstrated significant variations in individual-level variables. Most variables, excluding the MRI performance for patients with ischemic stroke and the target year variable, displayed significant differences. Significant differences were observed in all hospital-level variables, with 24.1% and 5.9% of the patients with hemorrhagic and ischemic stroke, respectively, undergoing interventional treatment in the segment with a low number of neurologists (p < .0001). The general characteristics of each medical institution, not an individual, are presented in S2 Table.

**Table 1:** General characteristics of the study population.

Variables	Therapy methods													
	Hemorrhagic stroke						*P-value*	Ischemic stroke						*P-value*
	Total		Conservative		Intervention			Total		Conservative		Intervention		
	N	%	N	%	N	%		N	%	N	%	N	%	
	14,254	100.0	7,668	53.8	6,586	46.2		46,407	100.0	39,078	84.2	7,329	15.8	
***Individual level***														
**Sex**							<.0001							<.0001
Men	7,111	49.9	4,080	57.4	3,031	42.6		27,168	52.8	22,636	83.3	4,532	16.7	
Women	7,143	50.1	3,588	50.2	3,555	49.8		19,239	37.4	16,442	85.5	2,797	14.5	
**Age**							<.0001							<.0001
18-44	1,520	10.7	703	46.3	817	53.8		1,622	3.2	1,336	82.4	286	17.6	
45-64	6,091	42.7	2,948	48.4	3,143	51.6		13,416	26.1	11,294	84.2	2,122	15.8	
65-79	4,245	29.8	2,342	55.2	1,903	44.8		18,743	36.4	15,485	82.6	3,258	17.4	
80-	2,398	16.8	1,675	69.8	723	30.2		12,626	24.5	10,963	86.8	1,663	13.2	
**Social security**							0.0016							<.0001
Health insurance	13,179	92.5	7,040	53.4	6,139	46.6		42,598	82.8	35,778	84.0	6,820	16.0	
Medical aid	1,075	7.5	628	58.4	447	41.6		3,809	7.4	3,300	86.6	509	13.4	
**Clinical subject**							<.0001							<.0001
Neuro-surgery	13,887	97.4	7,329	52.8	6,558	47.2		7,547	14.7	5,711	75.7	1,836	24.3	
Neurology	367	2.6	339	92.4	28	7.6		38,860	75.5	33,367	85.9	5,493	14.1	
**Lengths of stay**							<.0001							<.0001
-14	6,421	45.0	4,600	71.6	1,821	28.4		36,172	70.3	31,555	87.2	4,617	12.8	
15-28	4,485	31.5	1,953	43.5	2,532	56.5		6,677	13.0	5,099	76.4	1,578	23.6	
29-	3,348	23.5	1,115	33.3	2,233	66.7		3,558	6.9	2,424	68.1	1,134	31.9	
**Computed Tomography**							<.0001							<.0001
Yes	13,495	94.7	7,088	52.5	6,407	47.5		34,946	67.9	28,180	80.6	6,766	19.4	
No	759	5.3	580	76.4	179	23.6		11,461	22.3	10,898	95.1	563	4.9	
**Magnetic Resonance Imaging**							<.0001							0.2557
Yes	4,013	28.2	2,412	60.1	1,601	39.9		27,979	54.4	23,654	84.5	4,325	15.5	
No	10,241	71.8	5,256	51.3	4,985	48.7		18,428	35.8	15,474	84.0	2,954	16.0	
**Results**							0.0219							<.0001
Discharge	8,740	61.3	4,773	54.6	3,967	45.4		37,437	72.7	32,336	86.4	5,101	13.6	
Transfer	3,215	22.6	1,711	53.2	1,504	46.8		7,729	15.0	5,934	76.8	1,795	23.2	
Death	2,299	16.1	1,184	51.5	1,115	48.5		1,241	2.4	808	65.1	433	34.9	
**Charlson Comorbidity index**							<.0001							<.0001
0-1	9,158	64.2	5,200	56.8	3,958	43.2		32,672	63.5	28,172	86.2	4,500	13.8	
2	4,008	28.1	1,720	42.9	2,288	57.1		8,496	16.5	6,638	78.1	1,858	21.9	
≥ 3	1,088	7.6	748	68.8	340	31.3		5,239	10.2	4,268	81.5	971	18.5	
**Year**							0.2768							0.0524
2018	7,347	51.5	3,920	53.4	3,427	46.6		22,150	43.0	18,728	84.6	3,422	15.4	
2021	6,907	48.5	3,748	54.3	3,159	45.7		24,257	47.1	20,350	83.9	3,907	16.1	
***Hospital level***														
**Number of neurosurgery doctor**							<.0001							<.0001
Q1 (low)	1,280	9.0	972	75.9	308	24.1		5,465	11.8	5,145	94.1	320	5.9	
Q2	2,386	16.7	1,249	52.3	1,137	47.7		7,151	15.4	5,958	83.3	1,193	16.7	
Q3 (high)	10,588	74.3	5,447	51.4	5,141	48.6		33,791	72.8	27,975	82.8	5,816	17.2	
**Medical institution type**							<.0001							<.0001
Tertiary general hospital	6,139	43.1	3,082	50.2	3,057	49.8		20,025	43.2	16,486	82.3	3,539	17.7	
General hospital	8,115	56.9	4,586	56.5	3,529	43.5		26,382	56.8	22,592	85.6	3,790	14.4	
**Institution establishment type**							<.0001							<.0001
National·Public·Military hospital	938	6.6	609	64.9	329	35.1		2,914	6.3	2,575	88.4	339	11.6	
Private hospital	2,168	15.2	1,167	53.8	1,001	46.2		8,643	18.6	7,197	83.3	1,446	16.7	
Corporate hospital	11,148	78.2	5,892	52.9	5,256	47.1		34,850	75.1	29,306	84.1	5,544	15.9	
**Emergency medical center type**							<.0001							<.0001
Regional emergency medical center	5,678	39.8	2,933	51.7	2,745	48.3		17,827	38.4	14,683	82.4	3,144	17.6	
Local emergency medical center	7,219	50.6	3,826	53.0	3,393	47.0		23,109	49.8	19,464	84.2	3,645	15.8	
Local emergency medical agency	1,357	9.5	909	67.0	448	33.0		5,471	11.8	4,931	90.1	540	9.9	
**Hospital region**							0.0053							<.0001
Metropolitan	5,619	39.4	3,072	54.7	2,547	45.3		18,067	38.9	15,398	85.2	2,669	14.8	
Urban	4,370	30.7	2,262	51.8	2,108	48.2		14,275	30.8	11,945	83.7	2,330	16.3	
Rural	4,265	29.9	2,334	54.7	1,931	45.3		14,065	30.3	11,735	83.4	2,330	16.6	
**Quality evaluation grade**							<.0001							<.0001
Grade 1	12,645	88.7	6,561	51.9	6,084	48.1		40,610	87.5	33,729	83.1	6,881	16.9	
Etc	1,609	11.3	1,107	68.8	1,107	68.8		5,797	12.5	5,349	92.3	448	7.7	
**Hospital beds**							<.0001							<.0001
-300 beds	604	4.2	427	70.7	177	29.3		2,398	5.2	2,219	92.5	179	7.5	
301-600 beds	3,172	22.3	1,858	58.6	1,314	41.4		10,337	22.3	8,953	86.6	1,384	13.4	
601-900 beds	4,321	30.3	2,267	52.5	2,054	47.5		13,186	28.4	11,051	83.8	2,135	16.2	
901-1,200 beds	4,522	31.7	2,284	50.5	2,238	49.5		14,246	30.7	11,590	81.4	2,656	18.6	
1,200 beds-	1,635	11.5	832	50.9	803	49.1		6,240	13.4	5,265	84.4	975	15.6	
**Number of stroke patients**							<.0001							<.0001
< 200	2,612	18.3	1,639	62.7	973	37.3		8,862	19.1	8,011	90.4	851	9.6	
< 400	5,120	35.9	2,658	51.9	2,462	48.1		15,829	34.1	13,210	83.5	2,619	16.5	
< 600	4,107	28.8	2,150	52.3	1,957	47.7		13,347	28.8	11,016	82.5	2,331	17.5	
≥ 600	2,415	16.9	1,221	50.6	1,194	49.4		8,369	18.0	6,841	81.7	1,528	18.3	
**Number of neurology doctor**							<.0001							<.0001
0-2	2,897	20.3	1,763	60.9	1,134	39.1		9,209	19.8	8,094	87.9	1,115	12.1	
3-4	1,526	10.7	818	53.6	708	46.4		4,934	10.6	4,215	85.4	719	14.6	
5-6	1,576	11.1	831	52.7	745	47.3		4,895	10.5	4,047	82.7	848	17.3	
7-10	4,290	30.1	2,273	53.0	2,017	47.0		13,196	28.4	11,114	84.2	2,082	15.8	
11-15	2,487	17.4	1,217	48.9	1,270	51.1		8,326	17.9	6,727	80.8	1,599	19.2	
16-	1,478	10.4	766	51.8	712	48.2		5,847	12.6	4,881	83.5	966	16.5	

Results of the multilevel logistic regression analysis for interventional treatment suggested that the random effect (random variance) of the null model (baseline model), excluding the individual- and hospital-level independent variables, was 0.331 and 0.733, respectively, representing the variability among hospitals. Furthermore, the intraclass correlation coefficients, which indicate the proportion of variance at the hospital level to the total variance, were 9.14% and 18.23%, respectively. Thus, applying a multilevel analysis reflecting the hospital-level characteristics is appropriate (Tables 2 and 3). In Model 3, which included both individual- and hospital-level variables, the odds of undergoing interventional treatment were 60% lower in the hemorrhagic stroke group with fewer neurosurgeons than in the group with more neurosurgeons (odds ratio [OR] 0.40, 95% confidence interval [CI] 0.31–0.52) (Table 2). Similarly, for patients with ischemic stroke, the likelihood of receiving interventional treatment was 49% lower in the group with fewer neurosurgeons than in the group with more neurosurgeons (OR 0.51, 95% CI 0.39–0.65) (Table 3).

**Table 2:** Adjusted odds ratio of intervention by characteristics of individual- and hospital- level, multi-level model among hemorrhagic stroke patients.

Variables	Intervention							
	Null Model		Model 1		Model 2		Model 3	
	OR	95% CI	OR	95% CI	OR	95% CI	OR	95% CI
***Individual level(Fixed effect)***								
**Sex**								
Men			1.00				1.00	
Women			1.43(1.32–1.54)				1.42(1.31–1.53)	
**Age**								
18-44			3.06(2.62–3.58)				2.93(2.51–3.43)	
45-64			2.76(2.45–3.10)				2.65(2.35–2.98)	
65-79			2.08(1.84–2.34)				2.02(1.79–2.28)	
80-			1.00				1.00	
**Social security**								
Health insurance			1.27(1.10–1.47)				1.24(1.07–1.43)	
Medical aid			1.00				1.00	
**Clinical subject**								
Neuro-surgery			8.92(5.82–13.66)				8.61(5.61–13.21)	
Neurology			1.00				1.00	
**Lengths of stay**								
-14			1.00				1.00	
15-28			4.13(3.75–4.54)				4.19(3.80–4.60)	
29-			7.52(6.74–8.40)				7.65(6.85–8.54)	
**Computed Tomography**								
Yes			2.74(2.22–3.39)				2.76(2.24–3.41)	
No			1.00				1.00	
**Magnetic Resonance Imaging**								
Yes			0.61(0.56–0.67)				0.62(0.57–0.69)	
No			1.00				1.00	
**Treatment outcome**								
Discharge			1.00				1.00	
Transfer			0.76(0.68–0.84)				0.71(0.64–0.79)	
Death			2.27(2.02–2.55)				2.31(2.05–2.59)	
**Charlson Comorbidity index**								
0-1			1.00				1.00	
2			1.57(1.44–1.72)				1.57(1.44–1.72)	
≥ 3			0.44(0.37–0.51)				0.44(0.37–0.51)	
**Year**								
2018			1.00				1.00	
2021			1.03(0.95–1.12)				1.01(0.93–1.10)	
***Hospital level(Random effect)***								
**Number of neurosurgery doctor**								
Q1 (low)					0.37(0.29–0.47)		0.40(0.31–0.52)	
Q2					0.96(0.82–1.13)		1.03(0.86–1.24)	
Q3 (high)					1.00		1.00	
**Medical institution type**								
Tertiary general hospital					1.28(1.06–1.54)		1.70(1.37–2.11)	
General hospital					1.00		1.00	
**Institution establishment type**								
National·Public·Military hospital					1.00		1.00	
Private hospital					1.05(0.78–1.42)		1.36(0.97–1.92)	
Corporate hospital					1.08(0.85–1.36)		1.08(0.83–1.41)	
**Emergency medical center type**								
Regional emergency medical center					1.08(0.84–1.39)		1.08(0.82–1.44)	
Local emergency medical center					1.12(0.91–1.37)		1.19(0.94–1.49)	
Local emergency medical agency					1.00		1.00	
**Hospital region**								
Metropolitan					1.02(0.87–1.20)		1.12(0.93–1.35)	
Urban					1.01(0.84–1.20)		0.95(0.78–1.16)	
Rural					1.00		1.00	
**Quality evaluation grade**								
Grade 1					1.50(1.25–1.80)		1.35(1.09	–1.66)
Etc					1.00		1.00	
**Hospital beds**								
-300 beds					1.00		1.00	
301-600 beds					1.37(1.03–1.81)		1.32(0.96–1.80)	
601-900 beds					1.27(0.91–1.78)		1.35(0.93–1.98)	
901-1,200 beds					1.21(0.82–1.78)		1.24(0.80–1.92)	
1,200 beds-					1.14(0.71–1.84)		1.23(0.71–2.12)	
**Number of stroke patients**								
< 200					1.16(0.87–1.55)		1.06(0.76–1.46)	
< 400					1.01(0.81–1.27)		0.96(0.74–1.24)	
< 600					1.01(0.84–1.22)		1.04(0.83–1.29)	
≥ 600					1.00		1.00	
**Number of neurology doctor**								
0-2					1.19(0.85–1.67)		1.27(0.87–1.87)	
3-4					1.30(0.94–1.79)		1.34(0.92–1.94)	
5-6					1.10(0.82–1.47)		1.19(0.85–1.66)	
7-10					1.06(0.83–1.37)		1.01(0.76–1.34)	
11-15					1.19(0.96–1.48)		1.09(0.85–1.39)	
16-					1.00		1.00	
**Between area variance (SE)**	0.331(0.062)^*^		0.360(0.065)^*^		0.103(0.022)^*^		0.138(0.028)^*^	
**Percentage change in variation**			8.76%		68.88%		58.31%	
**Model Fitness**								
-2 Log Likelihood	19420.64		16421.48		19188.24		16199.5	
AIC	19424.64		16455.48		19236.24		16277.5	
**Intraclass correlation coefficient (%)**		9.14^a^						

adj.OR, adjusted odds ratio, CI, confidence interval, SE, standard error.

* p < .0001.

**Table 3:** Adjusted odds ratio of intervention by characteristics of individual- and hospital- level, multi-level model among ischemic stroke patients.

Variables	Intervention							
	Null Model		Model 1		Model 2		Model 3	
	OR	95% CI	OR	95% CI	OR	95% CI	OR	95% CI
***Individual level(Fixed effect)***								
**Sex**								
Men			1.00				1.00	
Women			0.85(0.80–0.90)				0.85(0.80–0.90)	
**Age**								
18-44			1.54(1.32–1.78)				1.51(1.30–1.76)	
45-64			1.41(1.30–1.53)				1.40(1.29–1.51)	
65-79			1.49(1.38–1.60)				1.48(1.38–1.59)	
80-			1.00				1.00	
**Social security**								
Health insurance			1.29(1.16–1.43)				1.27(1.15–1.42)	
Medical aid			1.00				1.00	
**Clinical subject**								
Neuro-surgery			3.70(3.34–4.10)				3.60(3.25–3.99)	
Neurology			1.00				1.00	
**Lengths of stay**								
-14			1.00				1.00	
15-28			1.97(1.83–2.12)				2.00(1.86–2.16)	
29-			2.80(2.56–3.06)				2.81(2.57–3.07)	
**Computed Tomography**								
Yes			5.38(4.88–5.94)				5.46(4.94–6.03)	
No			1.00				1.00	
**Magnetic Resonance Imaging**								
Yes			1.01(0.95–1.08)				1.01(0.94–1.07)	
No			1.00				1.00	
**Treatment outcome**								
Discharge			1.00				1.00	
Transfer			1.39(1.29–1.50)				1.37(1.27–1.47)	
Death			3.06(2.67–3.50)				3.04(2.65–3.48)	
**Charlson Comorbidity index**								
0-1			1.00				1.00	
2			1.71(1.59–1.83)				1.74(1.62–1.87)	
≥ 3			1.25(1.14–1.37)				1.25(1.15–1.37)	
**Year**								
2018			1.00				1.00	
2021			0.71(0.67–0.76)				0.73(0.68–0.78)	
***Hospital level(Random effect)***								
**Number of neurosurgery doctor**								
Q1 (low)					0.37(0.29–0.46)		0.51(0.39–0.65)	
Q2					0.89(0.77–1.04)		1.00(0.84–1.18)	
Q3 (high)					1.00		1.00	
**Medical institution type**								
Tertiary general hospital					1.14(0.94–1.38)		1.34(1.08–1.66)	
General hospital					1.00		1.00	
**Institution establishment type**								
National·Public·Military hospital					1.00		1.00	
Private hospital					1.03(0.72–1.46)		1.19(0.78–1.81)	
Corporate hospital					1.04(0.78–1.37)		1.19(0.85–1.67)	
**Emergency medical center type**								
Regional emergency medical center					1.33(1.02–1.72)		1.60(1.19–2.16)	
Local emergency medical center					1.35(1.09–1.66)		1.58(1.24–2.01)	
Local emergency medical agency					1.00		1.00	
**Hospital region**								
Metropolitan					0.93(0.76–1.15)		1.00(0.78–1.29)	
Urban					0.99(0.79–1.23)		1.04(0.80–1.36)	
Rural					1.00		1.00	
**Quality evaluation grade**								
Grade 1					1.81(1.49–2.19)		1.85(1.48–2.32)	
Etc					1.00		1.00	
**Hospital beds**								
-300 beds					1.00		1.00	
301-600 beds					1.49(1.11–2.00)		1.65(1.18–2.29)	
601-900 beds					1.02(0.71–1.46)		0.88(0.59–1.32)	
901-1,200 beds					1.07(0.70–1.63)		0.94(0.58–1.52)	
1,200 beds-					0.76(0.43–1.33)		0.65(0.34–1.26)	
**Number of stroke patients**								
< 200					0.56(0.42–0.75)		0.54(0.39–0.76)	
< 400					0.71(0.57–0.89)		0.65(0.50–0.84)	
< 600					0.90(0.76–1.05)		0.80(0.66–0.96)	
≥ 600					1.00		1.00	
**Number of neurology doctor**								
0-2					1.33(0.94–1.88)		0.53(0.36–0.80)	
3-4					1.09(0.79–1.51)		0.51(0.35–0.74)	
5-6					1.05(0.79–1.40)		0.70(0.51–0.96)	
7-10					0.93(0.73–1.19)		0.79(0.60–1.04)	
11-15					0.86(0.71–1.05)		0.80(0.65–0.99)	
16-					1.00		1.00	
**Between area variance (SE)**	0.733(0.108)^*^		1.211(0.161)^*^		0.264(0.042)^*^		0.436(0.066)^*^	
**Percentage change in variation**			65.21%		63.98%		40.52%	
**Model Fitness**								
-2 Log Likelihood	39439.81		35093.29		39118.61		34770.8	
AIC	39443.81		35127.29		39166.61		34848.8	
**Intraclass correlation coefficient (%)**		18.23^a^						

adj.OR, adjusted odds ratio, CI, confidence interval, SE, standard error.

* p < .0001.

Table 4 presents results of the subgroup analysis performed by subdividing the interventional treatment types. The interventional treatments were categorized into four groups: (1) surgery, (2) interventional radiology, (3) IV-tPA, and (4) concurrent interventional radiology and IV-tPA. A lower number of neurosurgeons was strongly associated with surgery (OR 0.42, 95% CI 0.32–0.56) and interventional radiology (OR 0.34, 95% CI 0.23–0.51) in patients with hemorrhagic stroke. In contrast, the odds of receiving treatment decreased in all categories, except surgery, in patients with ischemic stroke, specifically for interventional radiology (OR 0.31, 95% CI 0.21–0.45), IV-tPA (OR 0.59, 95% CI 0.46–0.77), and their simultaneous administration (OR 0.34, 95% CI 0.20–0.57). Therefore, for patients with hemorrhagic stroke, the number of surgeons was not associated with surgery. For patients with ischemic stroke, the number of surgeons was not associated with IV-tPA administration.

**Table 4:** Adjusted odds ratio of intervention type by characteristics of multi-level model among stroke patients.

Variables	Hemorrhagic stroke								Ischemic stroke							
	Surgery		Interventional Radiological Procedure		T-PA		Interventional Radiological Procedure & T-PA		Surgery		Interventional Radiological Procedure		T-PA		Interventional Radiological Procedure & T-PA	
	OR	95% CI	OR	95% CI	OR	95% CI	OR	95% CI	OR	95% CI	OR	95% CI	OR	95% CI	OR	95% CI
**Number of neurosurgery doctor**																
Q1 (low)	0.42 (0.32 – 0.56)		0.34 (0.23 – 0.51)		1.08 (0.42 – 2.79)		0.25 (0.02 – 3.62)		0.43 (0.17 – 1.13)		0.31 (0.21 – 0.45)		0.59 (0.46 – 0.77)		0.34 (0.20 – 0.57)	
Q2	0.84 (0.69 – 1.02)		1.04 (0.81 – 1.34)		0.93 (0.50 – 1.73)		0.78 (0.29 – 2.09)		1.03 (0.63 – 1.70)		1.15 (0.93 – 1.42)		1.01 (0.84 – 1.21)		1.33 (1.00 – 1.77)	
Q3 (high)	1.00		1.00		1.00		1.00		1.00		1.00		1.00		1.00	
**Between area variance (SE)**	0.171(0.032)^*^		0.387(0.073)^*^		0.770(0.192)^*^		0.434(0.245)^*^		0.681(0.168)^*^		0.713(0.112)^*^		0.215(0.039)^*^		0.397(0.080)^*^	
**Percentage change in variation**	22.27%		46.77%		29.74%		66.01%		108.26%		34.78%		27.85%		16.24%	
**Model Fitness**																
-2 Log Likelihood	15068.9		11630.26		3108.61		797.61		3002.39		23769.82		25641.36		11000.31	
AIC	14128.7		11708.26		3186.61		875.61		3080.39		23847.82		25719.36		11078.31	
**Intraclass correlation coefficient (%)**	6.27^a^		14.72^a^		24.99^a^		27.96^a^		9.04^a^		13.85^a^		8.31^a^		12.60^a^	

adj.OR, adjusted odds ratio, CI, confidence interval, SE, standard error.

* p < .0001

**Fig 1:**
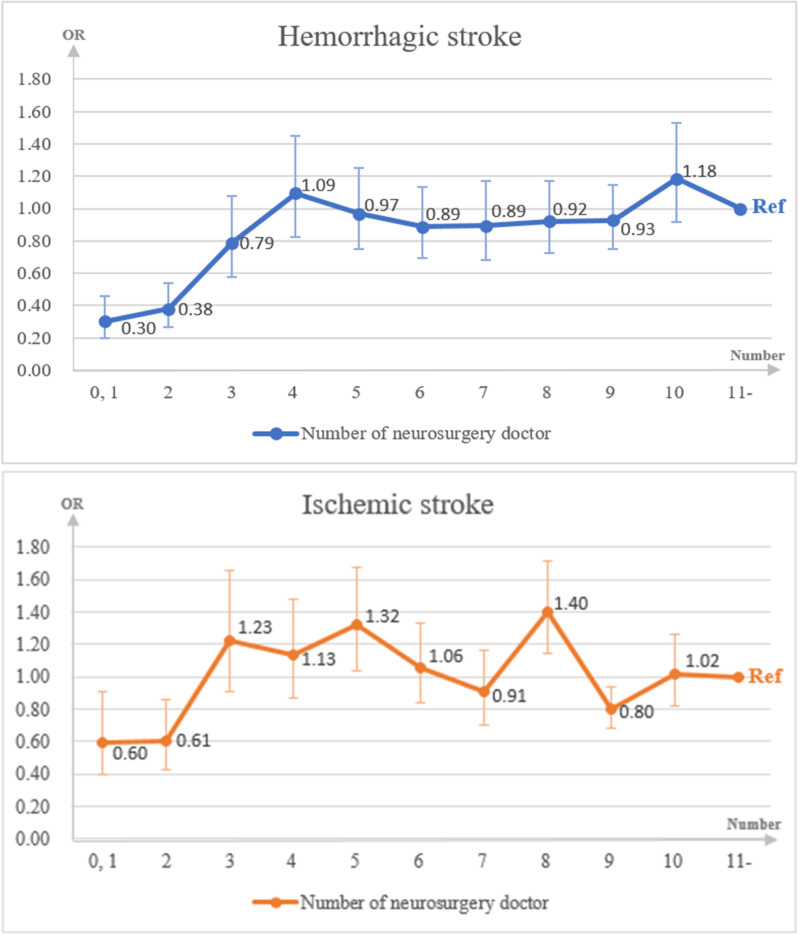
The odds ratio of interventional treatment based on the number of neurosurgeons in the multilevel model.

The odds ratio for receiving interventional treatment increased with three or more neurosurgeons for both patients with hemorrhagic (0–1: OR 0.30, 95% CI 0.20–0.46; 2: OR 0.38, 95% CI 0.27–0.54; and 3: OR 0.79, 95% CI 0.58–1.08) and ischemic (0–1: OR 0.60, 95% CI 0.39–0.91; 2: OR 0.61, 95% CI 0.43–0.86; and 3: OR 1.23, 95% CI 0.91–1.65) stroke (Fig 1). The probability of undergoing interventional treatment was significantly lower in groups with one or two neurosurgeons; the association became insignificant for groups with three or more neurosurgeons. Our results can serve as a predictive model for selecting an appropriate number of neurosurgeons.

## Discussion

We analyzed the impact of various individual- and hospital-level factors on the treatment of patients with acute stroke, categorizing them into hemorrhagic and ischemic stroke cases. Among the assessment variables for acute stroke, the probability of receiving interventional treatment in the first-grade group was higher for patients with hemorrhagic or ischemic stroke who were hospitalized and underwent interventional treatment. The probability of receiving interventional treatment for ischemic stroke in the first-grade group was higher for patients undergoing interventional radiology, IV-tPA administration, or a combination of both. This finding suggests the effectiveness of policies that consider the treatment history, including IV-tPA utilization and anticoagulant prescription rates, in the annual assessment of acute stroke conducted by the Assessment and Evaluation Agency. Research on mortality within 30 days between institutions that underwent the existing stroke appropriateness assessment and those that did not showed lower mortality rates in institutions that underwent the evaluation [25–27]. Therefore, results of the appropriateness assessment for acute stroke were confirmed to exert a significant impact on the interventional treatment and clinical outcomes [25–28].

Patients with ischemic stroke treated by neurosurgeons in the group that underwent fewer surgeries were more likely to receive interventional therapy than those in the group that underwent more surgeries. The probability of receiving interventional therapy was lower in the group with fewer neurosurgeons performing interventional radiology than in the group with more neurosurgeons. Patients treated by neurosurgeons with fewer instances of IV-tPA administration were more likely to undergo interventional therapy, compared with those treated by neurosurgeons with higher instances of IV-tPA administration. During simultaneous interventional radiology and IV-tPA administration, the probability of receiving interventional therapy was significantly higher in the group with fewer neurosurgeons than in the group with more neurosurgeons. Thus, we observed an association between the number of neurosurgeons and certain procedures (surgery, interventional radiological procedures, IV-tPA administration, and simultaneous interventional radiology and IV-tPA administration) in some subgroups of patients with ischemic stroke. For both patients with hemorrhagic and ischemic stroke, the group with fewer neurosurgeons demonstrated a lower probability of undergoing surgery and interventional radiology, highlighting the requirement for at least three neurosurgeons.

Finally, the number of neurosurgeons performing interventional therapy was disaggregated by unit and reanalyzed. In acute stroke cases, the probability of undergoing interventional therapy was higher when the number of neurosurgeons was three or more. Previous studies have not reported relevant data regarding the differences in treatment based on the number of neurosurgeons. A study on the number of neurologists for patients with stroke predicted and examined the number of neurologists based on the criteria for training neurologists in Korea, considering the predicted occurrence of stroke [29]. Furthermore, it suggested a shortage in the supply of neurologists owing to changes in the medical conditions and population structure by 2030. The support rate for neurosurgery residency programs ranged from 80% to 120% annually over the past 20 years [30]. However, an increasing trend in the dropout rate has been observed during the 4-year residency training, reaching 15.43% [30], and the number of graduates specializing in neurosurgery has decreased over time [30]. These findings indicate a potential shortage of neurosurgeons in the future, emphasizing the need for ongoing research and management strategies to ensure an adequate supply of neurosurgeons for patients who have had stroke [29,30].

Therefore, the treatment for patients with hemorrhagic and ischemic stroke was largely influenced by individual-level factors. Hospital-level factors, including the type of healthcare institution, appropriateness evaluation of acute stroke, and number of neurosurgeons, exerted some impact. The probability of performing interventional therapy is high when there are three or more neurosurgeons. The neurosurgeons included both specialists and residents, including those with licenses for neurosurgery. No previous studies exist on the differences in treatment based on the number of neurosurgeons treating patients with acute stroke. Existing studies have explored the impact of stroke on patient mortality, highlighting the urgent need for prompt treatment and adequate personnel for effective care [31].

### Limitations

However, this study has some limitations. First, we controlled for the factors influencing differences in the number of responsible doctors treating patients with acute stroke; however, we could not control all variables affecting patient treatment because of the inclusion of data based on healthcare claims. Second, we did not include social factors at the individual level and data that accurately reflected a patient's condition in a clinical setting, such as severity. Therefore, this drawback generated a high possibility of uncontrolled confounding variables. Third, we performed a cross-sectional study, suggesting an unclear temporal relationship and possible reverse causality. Furthermore, the anonymized nature of the dateset restricted our ability to conduct longitudinal analyses, as individual patient data could not be linked over time. Finally, our study was based solely on data from South Korea, and thus, the generalizability of our findings to other healthcare systems may be limited. Despite these limitations, this study has several strengths. First, in this novel study, we analyzed the treatments in detail by categorizing patients with acute stroke treated in tertiary and general hospitals into four types, accounting for approximately 90% of such patients in South Korea, and we performed a comprehensive analysis. Second, by recognizing the number of neurosurgeons treating acute stroke as a key variable, we provide fundamental data on the differences in treatment and the number of neurosurgeons responsible for interventional therapy. Finally, we conducted a multidimensional analysis of various individual- and hospital-level variables, ensuring their representativeness and reliability, by utilizing healthcare claims data from the Health Insurance Review and Assessment Service.

Future studies should aim to address these limitations by incorporating detailed clinical data, including stroke severity implementation of treatment during the golden time, and availability of specialized personnel. Longitudinal datasets would enable a more comprehensive analysis of causal relationships between neurosurgeon staffing levels and stroke outcomes. Additionally, expanding the scope of research to include data from other countries would allow for valuable international comparisons, providing a broader understanding of the relationship between neurosurgical resources and stroke care.

## Conclusion

The significance of this study lies in its analysis of the impact of the number of neurosurgeons on the treatment of patients with acute stroke, considering the high mortality and the ongoing need for continuous care because of the nature of stroke. Healthcare costs for patients with stroke continue to increase, characterized by high mortality and long-term disability. This study provides essential foundational data for healthcare policies. Our findings underscore the importance of having a minimum of three neurosurgeons to treat patients with acute stroke in Korea, suggesting a correlation between the number of medical professionals and the choice of treatment. This aspect highlights the need for ongoing research in essential medical fields directly related to the health and lives of citizens, focusing not only on the number of physicians but also on healthcare personnel, such as nurses, involved in severe and emergency care. In the long term, considering comprehensive factors, such as clinical reality, and developing efficient management and policy strategies for the allocation of personnel involved in stroke treatment will contribute to enhancing health equity among the population.

## Supporting information

S1 Fig Diagram of the study participant selection. (TIF)

S1 Table General characteristics of the medical institution. (DOCX)

S2 Table Distribution of intracerebral hemorrhage and subarachnoid hemorrhage in hemorrhagic stroke according to the intervention method. (DOCX)
