# Supplementary figures and images for: Differences in intervention for patients with acute stroke according to the manpower of neurosurgeons
Source: PLoS One. 2025 Mar 10;20(3):e0319740. doi: 10.1371/journal.pone.0319740 (PMC11892828; doi:10.1371/journal.pone.0319740)

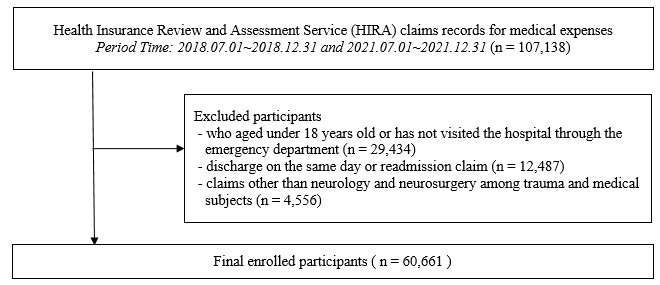

Supplement: S1 Fig — (TIF) [file pone.0319740.s001.tif]
