# Supplementary material for: Differences in intervention for patients with acute stroke according to the manpower of neurosurgeons
Source: PLoS One. 2025 Mar 10;20(3):e0319740. doi: 10.1371/journal.pone.0319740 (PMC11892828; doi:10.1371/journal.pone.0319740)
Supplement: S1 Table — (DOCX) [file pone.0319740.s002.docx]

| **S1 Table. General characteristics of the medical institution** | | | | |
| --- | --- | --- | --- | --- |
| **Variables** | **2018** | | **2021** | |
|  | **N** | **%** | **N** | **%** |
|  | 313 | 100.0 | 300 | 100.0 |
| **Medical institution type** |  |  |  |  |
| Tertiary general hospital | 42 | 13.4 | 45 | 15.0 |
| General hospital | 271 | 86.6 | 255 | 85.0 |
| **Institution establishment type** |  |  |  |  |
| National·Public·Military hospital | 57 | 18.2 | 39 | 13.0 |
| Private hospital | 57 | 18.2 | 61 | 20.3 |
| Corporate hospital | 199 | 63.6 | 200 | 66.7 |
| **Emergency medical center type** |  |  |  |  |
| Regional emergency medical center | 37 | 11.8 | 39 | 13.0 |
| Local emergency medical center | 115 | 36.7 | 123 | 41.0 |
| Local emergency medical agency | 161 | 51.4 | 138 | 46.0 |
| **Hospital region** |  |  |  |  |
| Metropolian | 105 | 33.5 | 98 | 32.7 |
| Urban | 92 | 29.4 | 94 | 31.3 |
| Rural | 116 | 37.1 | 108 | 36.0 |
| **Quality evaluation grade** |  |  |  |  |
| Grade 1 | 143 | 45.7 | 133 | 44.3 |
| Etc | 170 | 54.3 | 167 | 55.7 |
| **Hospital beds** |  |  |  |  |
| -300 beds | 91 | 29.1 | 82 | 27.3 |
| 301-600 beds | 132 | 42.2 | 128 | 42.7 |
| 601-900 beds | 47 | 15.0 | 47 | 15.7 |
| 901-1,200 beds | 31 | 9.9 | 31 | 10.3 |
| 1,200 beds- | 12 | 3.8 | 12 | 4.0 |
| **Number of stroke patients** |  |  |  |  |
| < 200 | 218 | 69.6 | 198 | 66.0 |
| < 400 | 55 | 17.6 | 62 | 20.7 |
| < 600 | 26 | 8.3 | 30 | 10.0 |
| ≥ 600 | 14 | 4.5 | 10 | 3.3 |
| **Number of neurology doctor** |  |  |  |  |
| 0-2 | 201 | 64.2 | 171 | 57.0 |
| 3-4 | 29 | 9.3 | 46 | 15.3 |
| 5-6 | 19 | 6.1 | 17 | 5.7 |
| 7-10 | 36 | 11.5 | 36 | 12.0 |
| 11-15 | 15 | 4.8 | 19 | 6.3 |
| 16- | 13 | 4.2 | 11 | 3.7 |
| **Number of neurosurgery doctor** |  |  |  |  |
| 0-2 | 169 | 54.0 | 150 | 50.0 |
| 3-4 | 56 | 17.9 | 61 | 20.3 |
| 5-6 | 37 | 11.8 | 38 | 12.7 |
| 7-10 | 33 | 10.5 | 32 | 10.7 |
| 11- | 18 | 5.8 | 19 | 6.3 |
