# Supplementary material for: Differences in intervention for patients with acute stroke according to the manpower of neurosurgeons
Source: PLoS One. 2025 Mar 10;20(3):e0319740. doi: 10.1371/journal.pone.0319740 (PMC11892828; doi:10.1371/journal.pone.0319740)
Supplement: S2 Table — (DOCX) [file pone.0319740.s003.docx]

| **S2 table. Distribution of intracerebral hemorrhage and subarachnoid hemorrhage in hemorrhagic stroke according to the intervention method** | | | | | | |
| --- | --- | --- | --- | --- | --- | --- |
| **Type of Hemorrhagic stroke** | **Total** | | **Intervention** | | | |
|  |  |  | **Endovascular treatment** | | **Craniotomy** | |
|  | **N** | **%** | **N** | **%** | **N** | **%** |
|  | 14,254 | 100.0 | 2,744 | 100.0 | 11,510 | 100.0 |
| Intracerebral haemorrhage | 8,741 | 61.3 | 82 | 3.0 | 8,659 | 75.2 |
| Subarachnoid haemorrhage | 4,732 | 33.2 | 2,649 | 96.5 | 2,083 | 18.1 |
| Others* | 781 | 5.5 | 13 | 0.5 | 768 | 6.7 |

* Others: Nontraumatic subdural or extradural haemorrhage
